# Design and Preparation of Biomass-Derived Activated Carbon Loaded TiO_2_ Photocatalyst for Photocatalytic Degradation of Reactive Red 120 and Ofloxacin

**DOI:** 10.3390/polym14050880

**Published:** 2022-02-23

**Authors:** Yousef Gamaan Alghamdi, Balu Krishnakumar, Maqsood Ahmad Malik, Sultan Alhayyani

**Affiliations:** 1 Chemistry Department, Faculty of Sciences, King Abdulaziz University, P.O. Box 80203, Jeddah 21589, Saudi Arabia; ygalghamdi@kau.edu.sa; 2 Environmental Science and Engineering Laboratory, Department of Civil Engineering, Yeungnam University, Geongsan 38541, Korea; 3 Department of Chemistry, College of Sciences & Arts, King Abdulaziz University, P.O. Box 344, Rabigh 21911, Saudi Arabia; salhayyani@kau.edu.sa

**Keywords:** biomass, degradation, Reactive Red 120, ofloxacin, photocatalysis

## Abstract

The design and development of novel photocatalysts for treating toxic substances such as industrial waste, dyes, pesticides, and pharmaceutical wastes remain a challenging task even today. To this end, a biowaste pistachio-shell-derived activated carbon (AC) loaded TiO_2_ (AC-TiO_2_) nanocomposite was fabricated and effectively utilized towards the photocatalytic degradation of toxic azo dye Reactive Red 120 (RR 120) and ofloxacin (OFL) under UV-A light. The synthesized materials were characterized for their structural and surface morphology features through various spectroscopic and microscopic techniques, including high-resolution transmission electron microscope (HR-TEM), field emission scanning electron microscope (FE-SEM) along with energy dispersive spectra (EDS), diffuse reflectance spectra (DRS), X-ray diffraction (XRD), Fourier-transform infrared spectroscopy (FTIR), X-ray photoelectron spectroscopy (XPS), Raman spectroscopy, photoluminescence spectra (PL) and BET surface area measurements. AC-TiO_2_ shows enhanced photocatalytic activity compared to bare TiO_2_ due to the change in the bandgap energy and effective charge separation. The degradation rate of dyes was affected by the bandgap of the semiconductor, which was the result of the deposition weight percentage of AC onto the TiO_2_. The presence of AC influences the photocatalytic activity of AC-TiO_2_ composite towards RR 120 and OFL degradation. The presence of heteroatoms-enriched AC enhances the charge mobility and suppresses the electron-hole recombination in AC-TiO_2_ composite, which enhances the photocatalytic activity of the composite. The hybrid material AC-TiO_2_ composite displayed a higher photocatalytic activity against Reactive Red 120 and ofloxacin. The stability of the AC-TiO_2_ was tested against RR 120 dye degradation with multiple runs. GC-MS analyzed the degradation intermediates, and a suitable degradation pathway was also proposed. These results demonstrate that AC-TiO_2_ composite could be effectively used as an ecofriendly, cost-effective, stable, and highly efficient photocatalyst.

## 1. Introduction

The massive levels of pollutants released into water sources worldwide due to increased population expansion and industrial development have generated adverse environmental effects in recent decades [1,2,3,4]. The effluents from textile industries, particularly synthetic dyes, discharged without proper treatment and management adversely affect our environment in one way or another [1]. Moreover, synthetic dyes, present in many industrial wastewaters, are highly stable molecules and are difficult to degrade by conventional physicochemical processes [2]. The dyes and antibiotics in the wastewater of pharmaceutical plants and industrial effluents are among the most serious and hazardous pollutants that must be removed from the wastewater before discharging [3]. These toxic and dangerous substances are harmful and cannot be removed through conventional methods such as physical adsorption and biochemical techniques [4]. Thus, a suitable alternative approach such as advanced oxidation processes (AOPs) has been developed [5,6,7,8,9]. Semiconductors illuminated with UV or solar light (or any other strong light source), causes a valence electron to move to the conduction band, leaving a hole in the valence band. However, there is a drawback: the separated electron-hole pairs recombine immediately in unmodified semiconductors and decrease the photoactivity of the materials. To overcome these problems, a suitable modification was carried out through doping and surface modifications with a metal, a nonmetal, a mixture with other semiconductors, and photostable organic compounds [10,11,12,13,14]. It is imperative to select a suitable semiconductor material to efficiently degrade dyes with good stability.

Presently, a rising trend in wastewater treatment research focuses on generating and releasing reactive oxygen species (ROS). Ali and co-workers demonstrated that graphene-SBA/TiO_2_ has anti-photoerosion potential and efficiently degrades an antibiotic drug (tetracycline) and MB (methylene blue) under light [15]. They concluded that graphene-SBA/TiO_2_ has better photocatalytic activity than SBA/TiO_2_. In the visible light region, the compositions of WOx and the crystallinity of g–C3N4 affect the performance of the heterostructures, with improved photocatalytic activity for degradation and H2 generation obtained on W18O49/g–C3N4 layered heterostructures [16]. In semiconductor photocatalysis, suppressing photogenerated e^−^h^+^ recombination is essential for efficient charge separation and photoactivity. The coupling of g-C_3_N_4_ nanosheets with WOx has been a prevalent method that is very active towards dye degradation and hydrogen production. The potential of technologies and improvements must be economically rational. Carbonaceous materials possess various advantages, including their surface area, favourable surface functionality, high chemical and thermal stability, environmentally feasible preparation methods, and cost-effective products [17,18,19,20,21,22,23,24]. The carbon derived from biomass mixed with metal/metal oxides/sulfides and other semiconductors possesses good photocatalytic activity [19]. Several carbon resources were used to prepare carbons in the past decades, such as fossil-based hydrocarbons (e.g., lignite), biomass (e.g., wood), and polymers. Most biomass-derived carbon-based materials were used for energy technologies such as supercapacitor applications, sensors, and hydrogen evolution reactions [20,24,25,26,27,28,29]. However, the photo and bioapplications of biomass-derived carbon-based materials are scarce [24]. However, graphene and activated carbon-based materials are exclusively utilized for photocatalytic applications [30,31]. Due to its highly promising qualities such as high photocatalytic effectiveness, sufficient chemical and biological stability, cost-effective synthesis, non-noxious nature, and long-term stability, titanium dioxide (TiO_2_) is an efficient and renowned photocatalyst [32,33,34]. Photocatalytic elimination of dangerous environmental contaminants mediated by TiO_2_ is a potential method for treating biorecalcitrant organic waste materials [32,33,34,35,36]. Moreover, the applications related to the removal of pollutants, bacterial disinfection, H_2_ production, CO_2_ reduction, sensors, supercapacitors, and other fields are well documented [37,38]. In this work, the biomass-derived carbon has been synthesized from biological waste such as pistachio shell (dry fruits shell) and effectively utilized for toxic chemical degradation along with TiO_2_. The photocatalytic activity of pistachio shell-derived activated carbon loaded TiO_2_ (AC-TiO_2_) has been investigated toward degradations of toxic dye RR 120 and ofloxacin using UV-A light under various experimental conditions.

## 2. Materials and Methods

### 2.1. Chemicals

The titania source from titanium isopropoxide (TiiP) and antibiotic ofloxacin (OFL) was obtained from Sigma Aldrich. Reactive Red 120 (RR 120) (Balaji Colour Company, Chennai, India) and gelatin (C_102_H_151_O_39_N_31_, Sino Pharm Chemical Reagent Co., Ltd., Shanghai, China) were used as received. Pistachio shells were collected from the local market. Double-distilled water (DI) was used for the preparation of dye and OFL solutions.

### 2.2. Synthesis of Pistachio Shell Derived Activated Carbon (AC)

**Scheme 1:**
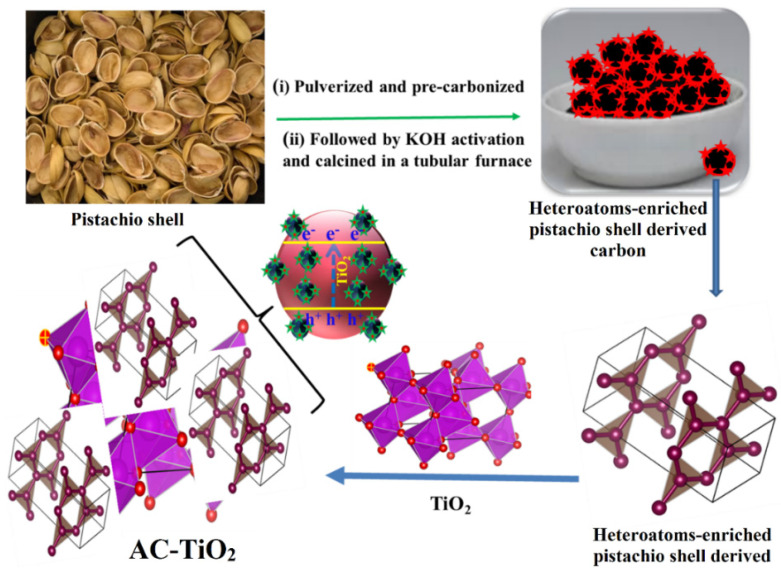
Schematic representation of the synthesis of AC and AC-TiO_2_.

Pistachio-shell-derived carbon (AC) was synthesized from pistachio shell (*Pistacia vera*), a biomass/biological waste. The prepared AC was activated by treating with KOH, and a schematic illustration is given as Scheme 1. In brief, the pistachio shells were dried under sunlight and then dried in an oven at 100 °C for 10 h. Then 20 g of pistachio shells were crushed well and pulverized. The pulverized shells were taken in a silica crucible, and precarbonized at 450 °C for 3 h in a hot air oven, then allowed to cool at room temperature. The black solids were collected and ground well with pestle and mortar. For activation, the black powder was treated with 12% KOH solution for 5 h at 90 °C. The activated slurry was filtered and placed in open air for a night and subsequently transferred into a silica crucible put into a tubular furnace at 800 °C for 3 h. Next, the AC was washed with HCl (5%) and double-distilled water until the pH became neutral and dried at 100 °C for 5 h to remove the moisture and smoothly ground using pestle and mortar to obtain fine AC powder.

### 2.3. Fabrication of AC-TiO_2_

Initially, TiO_2_ was synthesized by a sol-gel method. About 0.5 g of gelatin was dissolved in 5 mL of hot water. To this, 90 mL of a 2-propanol solution containing 10 mL of TiiP was added under stirring conditions and then put in an ultrasonication for an hour. The formed gel was continuously stirred for 24 h. The precipitate was filtered, washed with double distilled water (DI) and ethanol, air-dried for 24 h, and dried in a hot air oven at 100 °C for 4 h. The prepared white solid was thoroughly ground using pestle and mortar, then calcinated at 450 °C for 6 h in a muffle furnace. About 900 mg of TiO_2_ was dispersed in 25 mL of 2-propanol. Exactly 100 mg of AC was dispersed in 25 mL of a 2-propanol solution under sonication. The AC dispersion was added to TiO_2_ suspension under vigorous stirring, and the stirring continued overnight. The formed AC-TiO_2_ (Scheme 1) was filtered and dried in a hot air oven at 100 °C for 5 h. This catalyst contained ten wt % of AC on TiO_2_. The AC-TiO_2_ was also prepared by varying the deposition weight percentage (2.5, 5, 7.5, 10 wt %) of AC onto the fixed weight of as-prepared TiO_2_.

### 2.4. Characterization

X-ray diffraction (XRD) was used to examine the crystal structure of bare TiO_2_, AC, and AC-TiO_2_ utilizing a PANalytical X’Pert PRO powder X-ray diffractometer with 15KVA UPS support at two range of 10–80. Fourier-transform infrared spectroscopy (FT-IR) was used to investigate the surface functional groups of the materials (Thermofisher Scientific Nicolet iS5 spectrometer). A micro-Raman spectrometer, image spectrograph STR 500 mm focal length laser Raman spectrometer with flat field of 27 mm (W) 14 mm and (H) resolution of 1/0.6 cm^−1^/pixel was used to measure Raman spectra. UV-vis diffuse reflectance spectroscopy (UV-DRS) with Thermo Fisher Evaluation was used to examine the optical characteristics of the samples in the 190 to 1100 nm range. A Varian Cary eclipse photoluminescence spectrophotometer was used to record PL spectra with an extremely low temperature LN2 77 K set up. The morphologies of the obtained products were examined using a field emission scanning electron microscope (FE-SEM) equipped with an energy-dispersive X-ray spectrum (EDS) and a high-resolution transmission electron microscope (HR-TEM) (FEI –TECNAI G2-20 TWIN with LaB6 filaments) operating at 200 kV. A Perkin-Elmer UV spectrometer lambda 35 was used to record the absorption spectra of all the samples and a (Systronics) digital pH meter was used to modify the pH values using H_2_SO_4_ or NaOH. The ESCA-3 Mark II spectrometer (VG Scientific Ltd., East Grinstead UK) was used to obtain XPS spectra, with Alka (1486.6 eV) radiation as the source and C1s (285 eV) as the reference. The specific surface area of AC was determined through BET nitrogen adsorption-desorption at 77 K using Autosorb-iQ 2ST/MP, Quantachrome. The GC-MS (Shimadzu QP2020-NX, Columbia, Maryland, United States) was utilized for GC-MS analysis since it directly connects to the capillary column. Temperature-programmed mode was used on the GC column, with temperatures ranging from 50 to 350 degrees Celsius. The injection port was heated to a maximum of 450 °C, and the carrier gas column flow rate was 15 mL/min. 

### 2.5. Photocatalytic Degradation of Reactive Red 120 and Ofloxacin

To evaluate the photocatalytic activity of the materials, RR 120 and OFL were taken as modal pollutants, and the degradation of these pollutants was carried out under UV-A light. The photocatalytic degradation of Reactive Red 120 (50 ppm, 100 mL) and ofloxacin (10 ppm, 100 mL) was performed using Heber multilamp photoreactor model: HML-COMPACT-LP-MP88. The aqueous solution of both Reactive Red 120 and ofloxacin was poured into a quartz tube with a diameter of 2.5 cm and a length of 37 cm. The photocatalytic experiments were carried out using known amount photocatalysts (50 mg) dispersed in 100 mL of 50 ppm Reactive Red 120 and 100 mL of 10 ppm ofloxacin solution respectively under four 8-W mercury UV lamps with a wavelength of 365 nm. A Perkin-Elmer UV-vis spectrometer was used to evaluate the absorbance of the filtered solution. A gas chromatography-mass spectrometer (GC-MS) system was used to make the intermediate identification of the products. The sample for (GC-MS) analysis was made by extracting a portion of a 50 mL irradiated solution (after removing AC-TiO_2_ particles) three times with 50 mL dichloromethane each time. A beaker was used to capture the organic layer (dichloromethane). To remove the H_2_O, anhydrous sodium sulfate was added, filtered, and then evaporated in the air. After evaporation, an HPLC grade methanol was added (1 or 2 mL) to the beaker, and the contents were shaken well, then the solution was subjected to GC-MS analysis. The photodegradability of AC-TiO_2_ with deposition of different weight percentages of AC onto TiO_2_ was also investigated UV light toward RR 120 and OFL degradation to achieve the optimum reaction condition for further photocatalytic analysis. The optimum wt % of AC on TiO_2_ was found to be 10 wt % with lowest bang gap energy.

## 3. Results and Discussion

### 3.1. Structural and Morphological Investigations

**Figure 1:**
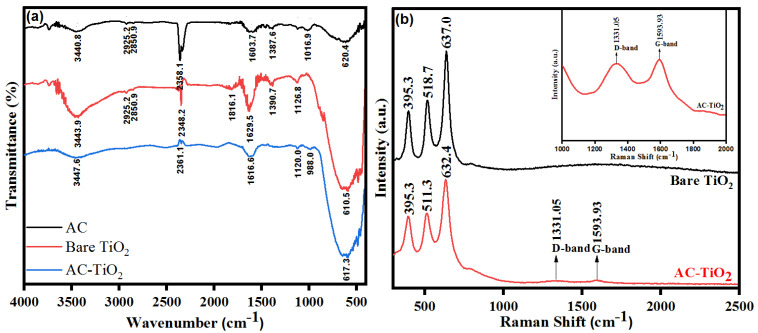
(**a**) FTIR of AC, bare TiO_2_ and AC-TiO_2_ and (**b**) Raman spectra of bare TiO_2_ and AC-TiO_2_.

The functional groups of AC, bare TiO_2_, and AC-TiO_2_ materials were determined by FT-IR spectroscopy (Figure 1a). The bands between 1400 and 450 cm^−1^ are associated with the characteristic vibrational modes of TiO_2_. The bands at 610 cm^−1^ and 1390 cm^−1^ are assigned to the Ti–O and Ti–O–Ti stretching, respectively, which are characteristic of TiO_2_. The strong –OH stretching (between 3300 and 3500 cm^−1^) observed in bare TiO_2_ and AC-TiO_2_ could be due to the moisture [39]. The FT-IR spectra of AC showed bands from 2850–2925 cm^−1^, that could be due to C–H stretching and a band at 1603 cm^−1^, probably due to the stretching vibrations of C=C. Bands between 1387 to 1016 cm^−1^ were observed, which could be due to C–O stretching and C=C bending corresponding to cellulose, lignin, and biopolymers, which are present in the pistachio shell biomass [40]. After modifying the AC with the TiO_2_, such vibrational bands in those regions were altered or not perfectly observed. Raman spectra of the prepared materials are shown in Figure 1b. As a nondestructive technique, Raman spectroscopy is an excellent tool for analyzing the structure and vibrational modes of crystalline material composites. Figure 1b shows the comparative Raman spectra of bare TiO_2_ and AC-TiO_2_ with four typical Raman bands, respectively. The bare TiO_2_ shows three predominant bands at 395.3(B_1g(1)_), 518.7 (A_1g_ + B_1_g_(2)_), and 637.0 cm^−1^ (E_g_) corresponding to the anatase phase of TiO_2_ [41]. As for the AC-TiO_2_ is considered, it also shows three characteristic Raman bands at 395.3, 511.3, and 632.4 cm^−1^ corresponding to the B_1g(1)_, A_1g_ + B_1g(2)_, and E_g_ modes of anatase, respectively. Additionally, in AC-TiO_2_ composite, the two characteristic Raman bands at 1331.05 cm^−1^ (D-band) ascribed to disordered SP^3^-defective carbon and 1593.93 cm^−1^ (G-band) assigned to ordered sp^2^-bonded carbon atoms, confirms the presence of carbon in AC-TiO_2_ composite [42]. It has been noted that there is no major difference in the Raman scattering patterns between AC-TiO_2_ composite and bare TiO_2_ takes place on AC deposition on bare TiO_2_ [43]. Although, in our previous report, the characteristic carbon G and D bands appeared at 1599.8 and1347.3 cm^−1^, respectively for Pd/C in TiO_2_-P25@Pd/C [44,45].

**Figure 2:**
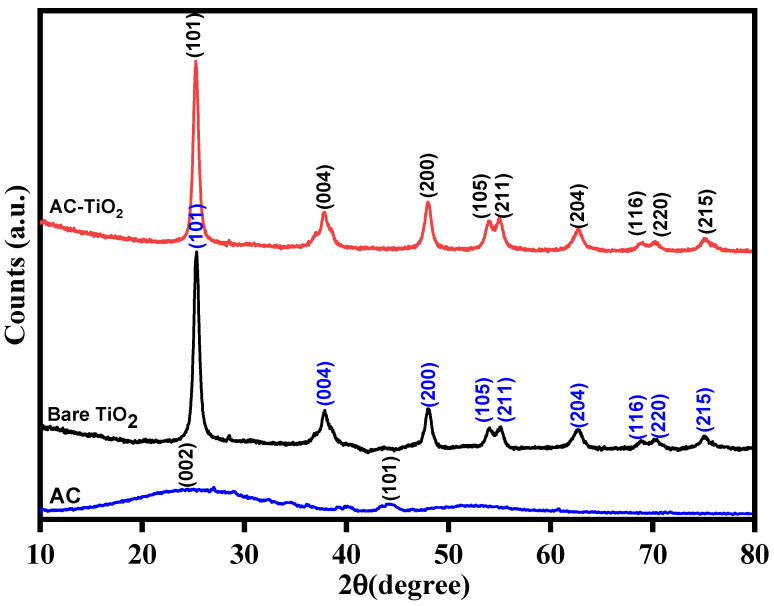
XRD pattern of AC, bare TiO_2_ and AC-TiO_2_.

XRD was applied to investigate the crystal phase of the synthesized materials. The XRD of AC, bare TiO_2_, and AC-TiO_2_ are presented in Figure 2. The pattern obtained for AC undoubtedly demonstrates its appearance in an amorphous state. The diffraction peaks appearing at 2ϴ values of 25.18°, 37.89°, 47.99°, 53.97°, 55.00°, 62.61°, 68.84°, 70.22° and 75.02° were indexed as (101), (004), (200), (105), (211), (204), (116), (220) and (215) and matched with the JCPDS card no. 21–1272 corresponds to anatase TiO_2_, signifying that, on calcination at 450 °C, the titania phase nucleates and consequently changes into nanocrystals [46]. The diffraction peaks obtained are distinct and sharp, with no shift in position, indicating that the prepared TiO_2_ has sufficient crystallinity and an organized framework. We found no secondary peaks associated with any other phase in the pure sample, showing that formed TiO_2_ only has an anatase phase. After the incorporation of AC with TiO_2_, there were no new peaks observed for AC in AC-TiO_2_, although AC shows two broad predominant peaks centered at 2ϴ values between 20 and 25, 40 and 45 belongs to (002) and (001) planes of carbon [47]. As can be seen, the peak strength remains unchanged when AC is introduced into TiO_2_, especially the primary peak (101) at 2θ = 43.3 for AC-TiO_2_ composite, showing that the crystallinity of TiO_2_ anatase phase is unaffected and that AC doping has no noticeable influence on TiO_2_ lattice organization. In addition, the diffraction pattern in all AC-TiO_2_ frameworks exhibited no peak corresponding to AC, showing that AC is either completely diffused in the bulk phase of AC-TiO_2_ or in an amorphous state [48]. The Debye–Scherrer equation (Equation (1)) was used to calculate the average crystallographic size of bare TiO_2_ and AC-TiO_2_ [49].

(1) $$D=\frac{K\lambda }{\beta \cos\theta }$$

In this equation, the crystal size of the catalyst (D) is calculated using diffraction angle (θ) and full width half maximum (FWHM) values (β). As we know, λ is the X-ray wavelength, and applying these values in the above equation, the average crystalline size of bare TiO_2_ is 12.0 nm. After AC modification of TiO_2_, the average crystalline size slightly decreased to 10.3 nm. The phase composition of AC–TiO_2_ is not changed and retained as bare TiO_2_, and possibly loading of AC occurred in the surface of TiO_2_.

**Figure 3:**
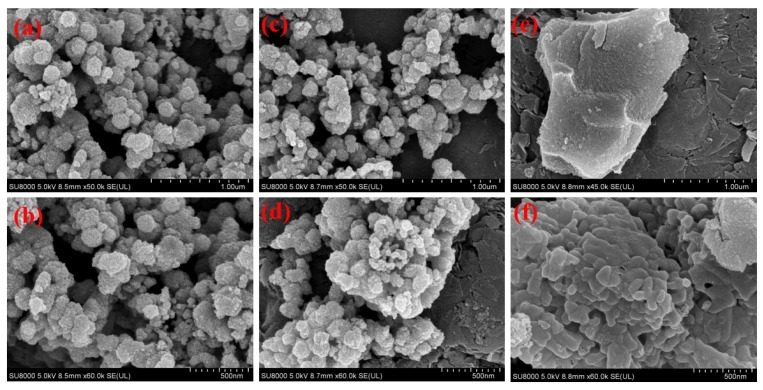
FE-SEM images of bare TiO_2_ (**a**,**b**), AC-TiO_2_ (**c**,**d**) and AC (**e**,**f**) at different magnifications.

The surface morphology of the bare TiO_2_, AC-TiO_2_, and AC were further analyzed by field emission scanning electron microscopy (FE-SEM) at different magnifications, as shown in Figure 3. However, the FE-SEM images of bare TiO_2_ displayed spherical and rough-surfaced particles with definite order distribution for the aggregated structures of individual fine nanosized particles (Figure 3a,b). The same type of morphology was observed when gelatine was used as a template for the gelatin assisted TiO_2_ [46]. To further characterize the morphology of AC-TiO_2_, the spheres obtained individual fine nanosized particles are clearly seen along with some of the AC particles (Figure 3c,d). In the case of pistachio-shell-derived activated carbon (AC), the particles are highly aggregated with irregular morphology particles (Figure 3e,f). Apparently, with the introduction of AC into TiO_2_, agglomeration of AC-TiO_2_ could be observed. This could lead to a dramatic change in photocatalytic properties.

**Figure 4:**
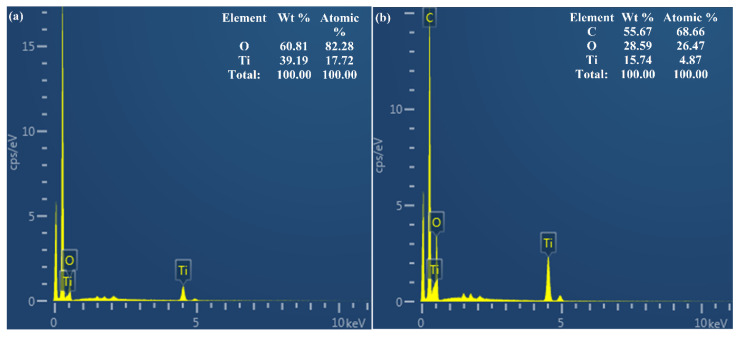
EDS of (**a**) bare TiO_2_ and (**b**) AC-TiO_2_.

**Figure 5:**
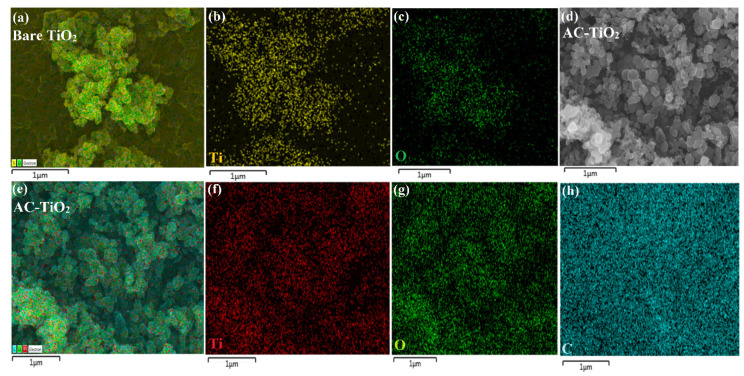
SEM and elemental mapping of bare TiO_2_ and AC-TiO_2_ nanomaterials.

The high magnification FE-SEM analysis further proves the presence of AC randomly on the surface of TiO_2_ microspheres and confirmed that the agglomeration occurred, but definite spheres are formed by aggregation of individual fine nanosized particles. The EDS and elemental mapping characterization were carried out to investigate the elemental composition of bare TiO_2_ and AC-TiO_2_ nanocomposites. The EDS spectra displayed that the synthesized bare TiO_2_ consisted of oxygen (O), and titanium (Ti), and AC-TiO_2_ nanocomposites consisted of titanium (Ti), oxygen (O), and carbon (C), as shown in Figure 4a,b. The mass fractions of O and Ti were 61.81% and 39.19%, respectively. In the case of AC-TiO_2_ nanocomposites, C, O, and Ti were 55.67%, 28.59%, and 15.74%, respectively. Meanwhile, the elemental mapping images of bare TiO_2_ revealed the presence and uniform distributions of Ti and O elements (Figure 5). The elemental mapping investigation of AC-TiO_2_ revealed the presence of Ti, O and confirms the uniform distribution of carbon, as shown in Figure 5.

**Figure 6:**
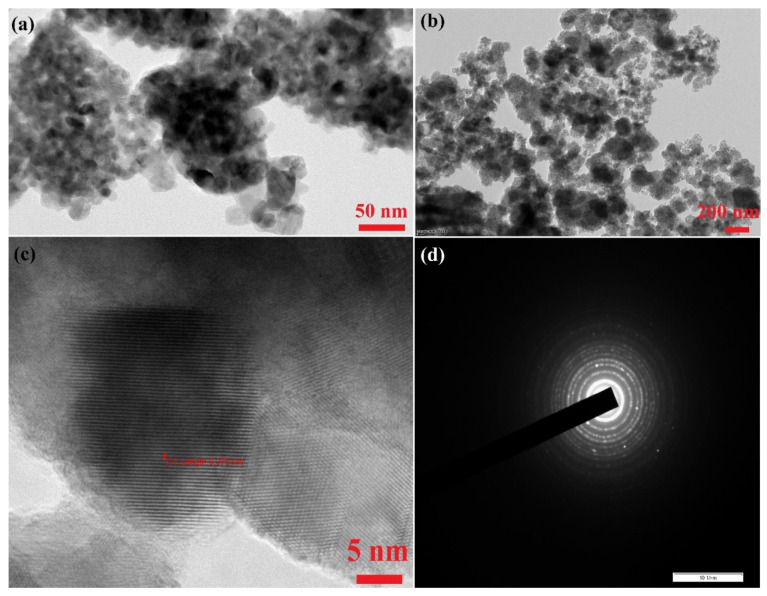
HR-TEM images of AC-TiO_2_ (**a**) 200 nm, (**b**) 50 nm, (**c**) high magnification TEM of AC-TiO_2_ (**d**) selected area electron diffraction (SAED) pattern of AC-TiO_2_.

To explore the morphology of AC-TiO_2_ nanostructured materials, the HR-TEM analysis at different magnifications was carried out, as shown in Figure 6a,b. The TEM analysis of AC-TiO_2_ illustrated that composite comprises almost regular shape nanoparticles grew with high density and little agglomeration. The size of these organized nanoparticles ranges from 10 to 30 nm. HR-TEM images also reveal that aggregating individual fine nanosized particles form definite spheres. Figure 6a,b confirms the successful nanocomposite creation between AC and TiO_2_. The HR-TEM image reveals that AC-TiO_2_ nanoparticles are firmly coordinated or under a pistachio shell biomass derived activated carbon (AC) blanket. High-resolution TEM analysis of AC-TiO_2_ shown in Figure 6c shows that the material exhibits good crystallization with d spacing or lattice spacing (distance between two adjacent planes) of about 0.370 nm, which is in good agreement with the (101) plane of TiO_2_ anatase phase. Additionally, the SAED pattern of AC-TiO_2_ nanocomposite shown in Figure 6d reveals that the materials are distinctly crystalline, which is also confirmed by the presence of highly ordered hollow centric rings of the SAED pattern.

**Figure 7:**
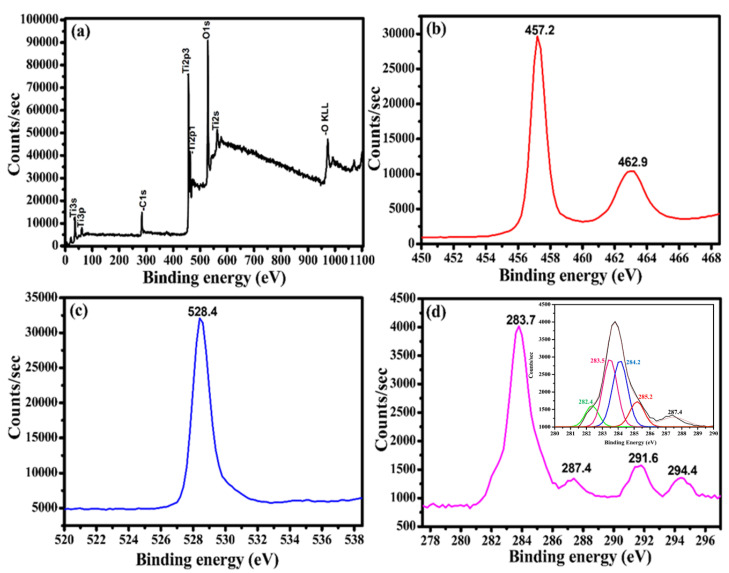
XPS spectra of AC-TiO_2_, (**a**) survey scan, and high-resolution XPS spectrum for (**b**) Ti2p_3_, (**c**) O1s, (**d**) C1s (inset- Deconvoluted XPS, C1 region of AC-TiO_2_).

XPS analysis was performed to acquire the surface oxidation states of AC-TiO_2_ nanocomposites, as shown in Figure 7. The survey spectrum of AC-TiO_2_ shown in Figure 7a reveals the presence of Ti, O, and C in the composite. The high-resolution XPS spectrum of Ti2p displayed in Figure 7b shows two prominent peaks at 457.2 eV and 462.9 eV, which are assigned to Ti2p doublets Ti 2p_3/2_ and Ti 2p_1/2_ that corresponds to Ti^4+^. These results confirmed that the Ti in AC-TiO_2_ has +4 primary valance state. The Ti 2p_3/2_ binding energy peak of pure TiO_2_ was observed between 458.0 and 458.5 eV [50,51]. The Ti 2p_3/2_ peak in AC-TiO_2_ observed binding energy is 457.2 eV, slightly lower (0.8 to 1.3 eV) than pure TiO_2_. The slight shift may be due to the presence of AC in AC-TiO_2_. The binding peak of O1s was observed at 528.4 eV, corresponding to lattice oxygen (O_L_) of TiO_2_ (Figure 7c). Four peaks were observed in the C1s region (Figure 7d) at 283.7, 287.4, 291.6, and 294.4 eV. Upon deconvolution, the peak at 283.7 eV was further divided into four peaks (Figure 7d(inset)) at 282.4, 283.5, 284.2 and 285.2 eV. In the C1 region, the peaks observed at 283.5, 284.2, 285.2, and 287.4 correspond to C−H, C−C/C=C, C−OH, and C=O, respectively [19,20]. The peak observed at 291.6 eV is ascribed to the Л−Л transition [52]. The origin of the peak at 282.4 eV was not clearly evidenced, but this may be due to heteroatom-doped carbons, which displayed a somewhat downshift field [53]. The unexpected peak at 294.4 eV (Figure 7d) may be originated from cations and/or oxides and/or metallic forms of residual potassium (K), and the K comes from the KOH activation process [54].

**Figure 8:**
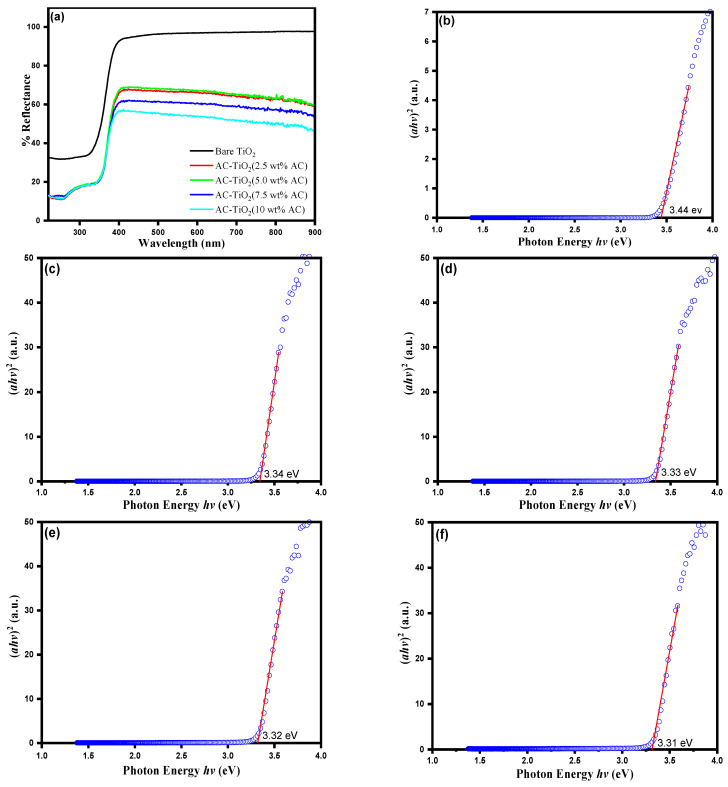
(**a**) UV-visible diffuse reflectance spectra of bare TiO_2_ and AC-TiO_2_, (**b**) Kubelka–Munk function versus light energy (*hv*) for band gap calculation of (**b**) bare TiO_2_, (**c**) AC-TiO_2_ (2.5 wt % AC), (**d**) AC-TiO_2_ (5 wt % AC), (**e**) AC-TiO_2_ (7.5 wt % AC), and (**f**) AC-TiO_2_ (10 wt % AC).

Diffuse reflectance UV–visible spectroscopy and PL studies were performed on the newly generated nanocomposites to explore the various optical aspects of the synthesized materials. For the semiconductor-based materials, the optical properties could be a quick study for investigating their bandgap and to know the ability to absorb UV-visible light. The solid-state UV-visible diffuse reflectance spectra (UV-Vis DRS) was taken for bare TiO_2_, and AC-TiO_2_ with different weight percentage AC deposition (2.5 wt %), (5 wt %), (7.5 wt %), and (10 wt %) and the results are shown in Figure 8a. The bandgap energy of bare TiO_2_ and AC-TiO_2_ with different weight percentage AC deposition was calculated by ${\left[F\left(R\right)h\nu \right]}^{1/2}$ using the Kubelka–Munk method [10] (Figure 8b–f). From these results, it was observed that AC-TiO_2_ (10 wt % AC) shows the lowest band gap compared to bare TiO_2_.

$$F\left(R\right)E^{1/2}={\left[\frac{{\left(1-R\right)}^{2}}{2R}\times h\nu \right]}^{1/2}$$

The optical bandgap energies of the bare TiO_2_, AC-TiO_2_ (2.5 wt % AC), AC-TiO_2_ (5 wt % AC), AC-TiO_2_ (7.5 wt % AC) and AC-TiO_2_ (10 wt % AC) were found to be 3.44 eV, 3.34 eV, 3.33 eV, 3.32 eV and 3.31 eV, respectively (Figure 8b–f). The optical bandgap of the AC-modified TiO_2_ was slightly lower than that of bare TiO_2_.

**Figure 9:**
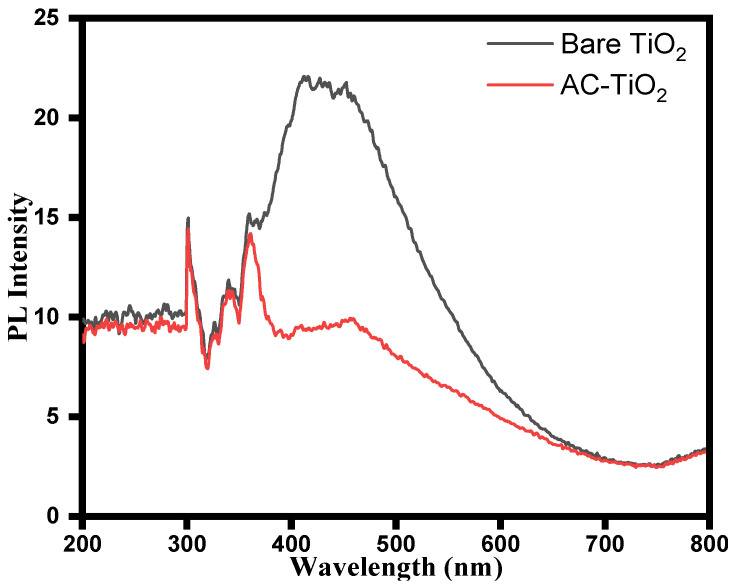
Photoluminescence spectra of bare TiO_2_ and AC-TiO_2_.

The photocatalytic excitation mechanism can be better understood by doing a PL study. To conduct a PL investigation, bare TiO_2_ and AC-TiO_2_ photocatalysts were tested at a wavelength of 320 nm, which is the excitation wavelength employed for PL studies. The photoluminescence spectra of photogenerated electrons and holes in the bare TiO_2_ and AC-TiO_2_ are shown in Figure 9. The intensity of the PL spectra determines the rate of electron-hole recombination; as the intensity is directionally proportional to the electron-hole recombination, the higher PL intensity reveals that the compound has a higher rate of electron-hole recombination, which is not favourable for photocatalytic applications. In order to get the excellent performance of the catalyst, it should have a lower electron-hole recombination rate. From this point of view, the maximum PL intensity was observed for bare TiO_2_ when compared with AC-TiO_2_. At 301, 343, 367, and 453 nm, bare TiO_2_ shows four prominent peaks. The peaks in the AC-TiO_2_ composite are virtually identical to those in bare TiO_2_, albeit with less intensity. The lower peak intensity could be owing to the presence of AC in the composites, which inhibits electron-hole recombination. The photocatalytic activity of AC-TiO_2_ may be particularly remarkable because of this reason.

**Figure 10:**
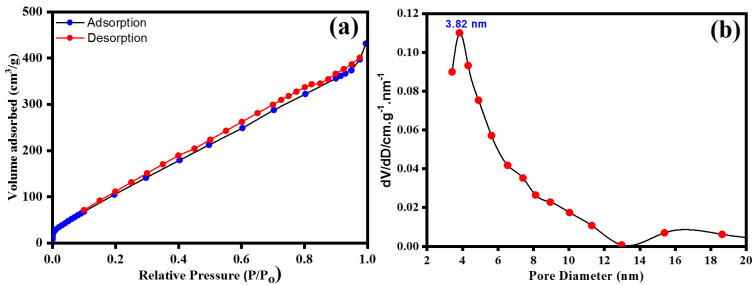
(**a**) BET nitrogen adsorption-desorption and (**b**) BJH pore size distribution curve of AC.

The N_2_ adsorption-desorption isotherm of AC is a typical type-IV isotherm with a hysteresis loop, indicating that the material synthesized is mesoporous (Figure 10a). AC has BET specific surface area (S_BET_) of 375.14 m^2^ g^−1^ and a pore volume of 0.52 cm^3^ g^−1^. According to the BJH pore size distribution study, the pore diameter of the synthesized AC is 3.82 nm (Figure 10b). The observed high specific surface area and high pore volume will facilitate electrolyte diffusion through the pores, allowing electrolytes to access the maximum surface area while decreasing the electron transport path. In addition, its high surface area improves catalyst dispersion and reduces sintering, resulting in higher catalytic efficiencies. Because of its large surface area, good adsorption capacity, inert and suitable pore structure, activated carbon has been investigated as a substrate for TiO_2_ photocatalysts [55].

### 3.2. Photocatalytic Investigations

**Figure 11:**
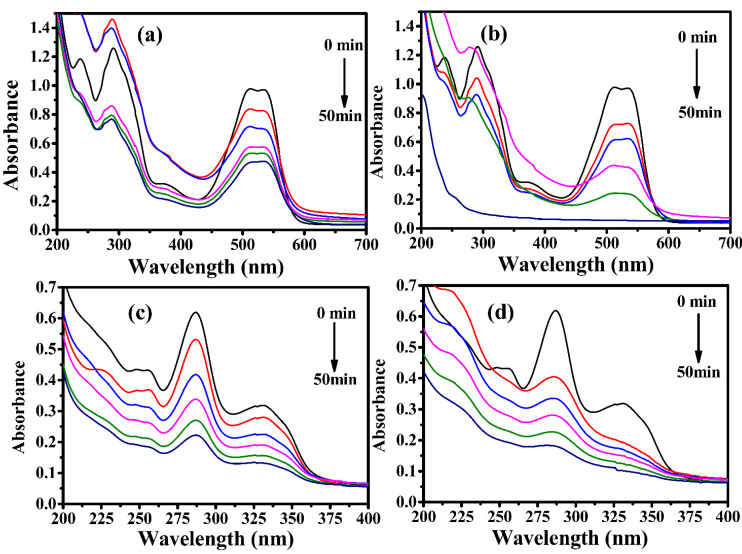
UV−vis spectra of RR 120 and OFL on irradiation with UV-A light (**a**) RR120-bare TiO_2_, (**b**) RR 120- AC-TiO_2_, (**c**) OFL-bare TiO_2_ and (**d**) OFL-AC-TiO_2_, pH = 5; [RR 120] = 50 ppm; [OFL] = 10 ppm; catalyst suspended = 50 mg/100 mL.

**Figure 12:**
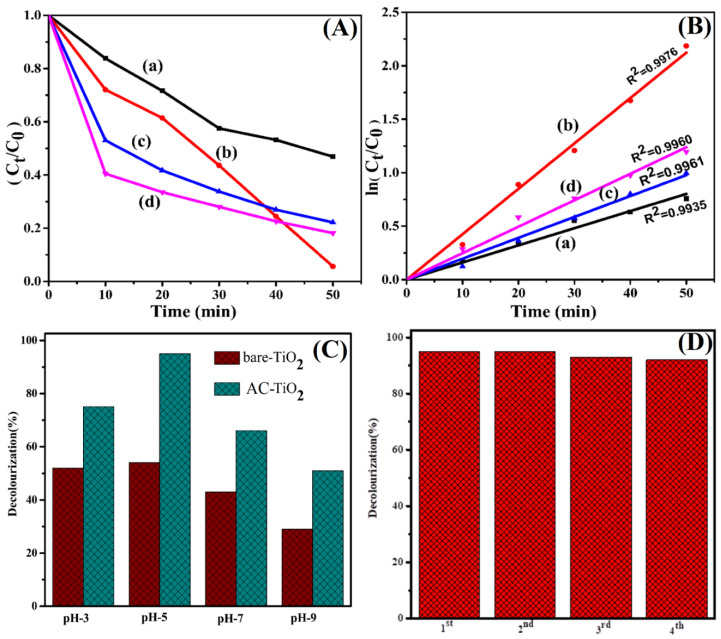
(**A**) Photodegradability of RR120 and OFL (**B**) corresponding kinetic data, (a) bare TiO_2_ (RR 120), (b) AC-TiO_2_ (RR 120), (c) bare TiO_2_ (OFL), (d) AC-TiO_2_ (OFL), pH=5; [RR 120] = 50 ppm; [OFL] = 10 ppm; Catalyst suspended = 50 mg/100 mL. (**C**) Effect of pH on RR 120 decolourization with UV-A light under bare TiO_2_ and AC-TiO_2_. [RR 120] = 50 ppm; catalyst suspended = 50 mg/100 mL. (**D**) Catalyst reusability on photocatalytic decolourization of RR 120 dye under UV A light, AC-TiO_2_ suspended = 50 mg/100 mL; pH = 5; irradiation time = 50 min.

The photocatalytic efficiency of bare TiO_2_ and AC-TiO_2_(10 wt % AC) was investigated using RR 120 and OFL as a model reaction under UV-light irradiation at an intensity of 225 mW.cm^−2^. The photocatalytic measurements were initially performed without the addition of bare TiO_2_ and AC-TiO_2_ photocatalysts, and changes in the absorbance of RR 120 and OFL were observed after exposure to UV-light for 50 min. In the second set of experiments, after adding bare TiO_2_ and AC-TiO_2_ photocatalysts to the RR 120 and OFL solution, the absorbance intensity of both RR 120 and OFL gradually decreases as the reaction proceeds (Figure 11). For RR 120 degradation, the dye was irradiated with AC-TiO_2_ gave 95% of degradation at 50 min, and under dark conditions without light, about 22% of dye adsorbed on the surface of the catalyst. At the same condition, bare TiO_2_ showed only 53% degradation under light. AC-TiO_2_ more effectively degraded the RR 120 dye than bare TiO_2_. The prepared materials activity was further evaluated by OFL degradation. About 82% of degradation occurred with AC-TiO_2_ at 50 min irradiation, and bare TiO_2_ gave a slightly lower value (77%) in the same irradiation time. These results confirm the excellent photocatalytic activity of AC-TiO_2_ compared to bare TiO_2_. Kinetic analysis of this photocatalytic reaction was performed based on the temporal decay of the RR 120 and OFL peaks, as shown in Figure 12A. The ratio C_t_/C_o_, where C_t_ and C_o_ are the RR 120 and OFL concentrations at time t and 0, was determined from the relative strength of the corresponding absorbance A_t_/A_o_. The corresponding kinetic values for Red 120 and ofloxacin degradation were calculated as shown in Figure 12B. The *pseudo*-first-order kinetic values of RR 120 degradation with AC-TiO_2_ and TiO_2_ were found to be 0.0424 and 0.0160 min^−1^, respectively. The corresponding OFL degradation kinetics values are 0.0247 and 0.0195 min^−1^. This reveals that the intermediates do not absorb at analytical wavelengths of 285 (RR 120), 512 nm (RR 120), and 288 nm (OFL).

For the photodegradation experiment, the role of the initial pH of the solution will highly influence the catalytic efficiency towards the degradation process. On the other hand, for the practical application, the effect of initial pH should be studied and discussed in a detailed way since the dye effluent comes from the dying industries in different pH. To consider this point, we examined the photodegradation RR 120 in different initial pH with bare TiO_2_ and AC-TiO_2_ under UV-light (Figure 12C). As the pH increased from 3 to 5, the percentage of degradation increased from 52 to 54 for bare TiO_2_ and 75 to 95 for AC-TiO_2_. Further increase of pH shows a decrease in activity observed for both cases. The influence of pH in the catalytic activity dominated more in AC-TiO_2_ composite than in bare TiO_2_. The point of zero charges (PZC) of TiO_2_ was reported to be 6.2 [56], and TiO_2_ had a positively charged surface below this value. At pH 7, RR 120 occurred as negative ions [10]. Because of the electrostatic attraction between positively charged TiO_2_ and negatively charged dye solution, an important contact between the dye and catalyst may occur, resulting in increased degradation [10].

The stability and the repeatability of the bare TiO_2_ and AC-TiO_2_ photocatalysts are the significant factors to investigate the feasibility of the prepared photocatalysts during the long-time irradiation. Therefore, the photocatalytic degradation of RR 120 by AC-TiO_2_ was recycled four times under the same experimental condition. The key role of any heterogeneous catalysis, especially for heterogeneous semiconductor photocatalysis towards water remediation, is the stability and reusability of the materials, which leads to a significant cost reduction of the effluent treatment. The reusability of the AC-TiO_2_ composite was checked towards RR 120 under UV-A light, and the results are combined in Figure 12D. The results showed a drop in efficiency from 95% (first turn) to 92% (fourth run). These results reveal that the AC-TiO_2_ composite was found to be stable and reusable for the degradation of RR 120 dye under UV-A light.

**Figure 13:**
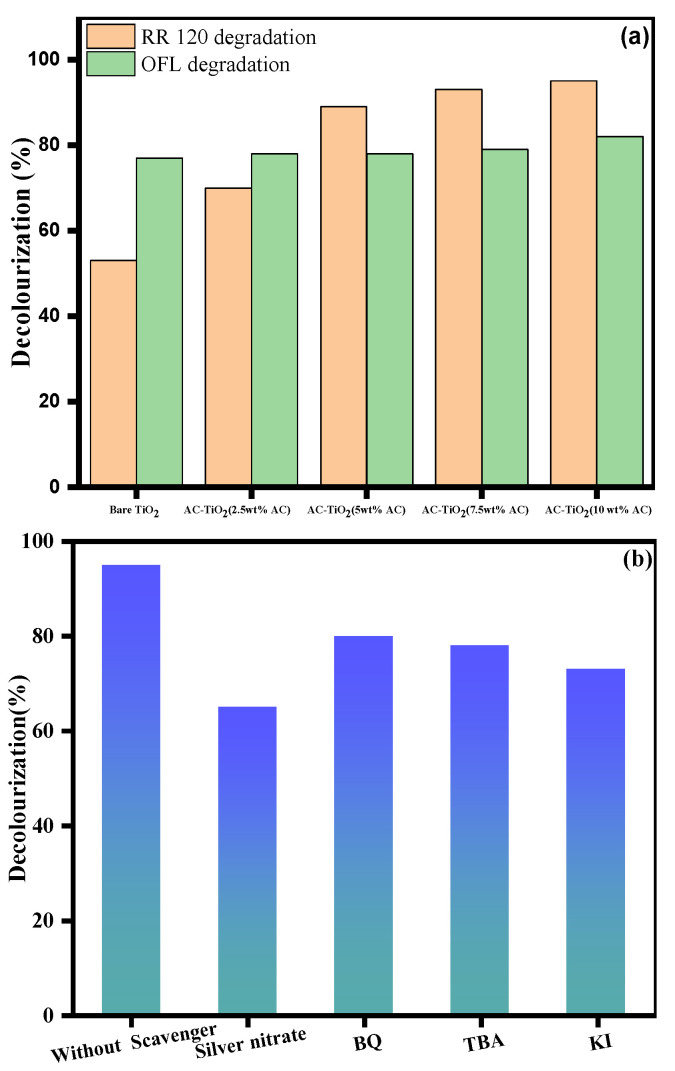
(**a**) Effect of AC deposition percentage on photocatalytic dye degradation of RR 120 and OFL (AC-TiO_2_ suspended = 50 mg/100 mL; pH = 5; irradiation time = 50 min), (**b**) photodegradability of RR 120 with and without scavenger, AC-TiO_2_ suspended = 50 mg/100 mL; pH = 5; irradiation time = 50 min.

The photocatalytic degradation reactions were also investigated for different AC dosage deposition on TiO_2_ having different band gap values (Figure 13a). Bare TiO_2_ shows only 53% and 77% degradation for RR 120 dyes and OFL, respectively. However, the degradation efficiency increased on increasing the AC deposition percentage on TiO_2_, which means that the increase in the AC deposition percentage resulted in the reduction of bandgap and inhibited the recombination rate

**Scheme 2:**
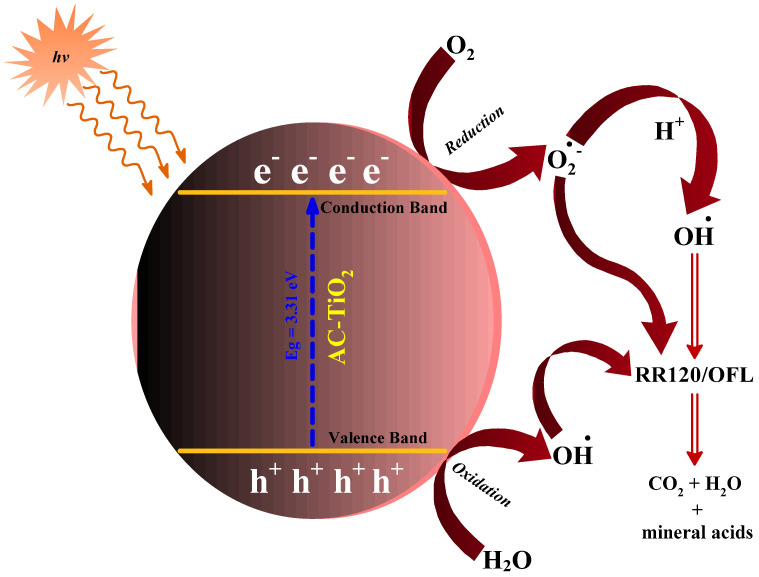
Mechanism of degradation of RR120 and OFL with AC-TiO_2_.

To further understand the photocatalytic mechanism of the as-prepared photocatalysts, the degradation mechanism of RR 120 and OFL by AC-TiO_2_ under UV-A light is shown in Scheme 2. When the TiO_2_ is irradiated with UV-A light, a valance electron goes to the conduction band (CB) and leaves a hole in the valence band (VB). The heteroatom-rich biomass-derived AC-TiO_2_ acts as an adsorbent and an electron mediator and suppresses the electron-hole recombination. The photo-excited electrons (CB) reacted with dissolved oxygen, producing superoxide radical anions (O_2_^●−^). In the meantime, hydroxyl radicals (^●^OH) were formed because of the reaction between holes (VB) and water, and both species were effective towards photocatalytic degrading of RR 120 and OFL. The scavenger experiment was performed in RR 120 degradation with AC-TiO_2_ under UV-A light to find the most active species involved in the photodegradation process of RR 120 and OFL (Figure 13b). The addition of 0.1 mmol of tertiary butyl alcohol (TBA, scavenger of ^●^OH), potassium iodide (KI, scavenger of h^+^), benzoquinone (BQ, scavenger of O_2_^●−^), and silver nitrate (AgNO_3_, scavenger of e^−^) contributed a significant decrease in the photodegradation efficiency of the catalyst. The degradation efficiency of the catalyst was dramatically reduced by the addition of silver nitrate, leading to the conclusion that electrons (e^−^) constitute the primary active species in the degradation.

**Scheme 3:**
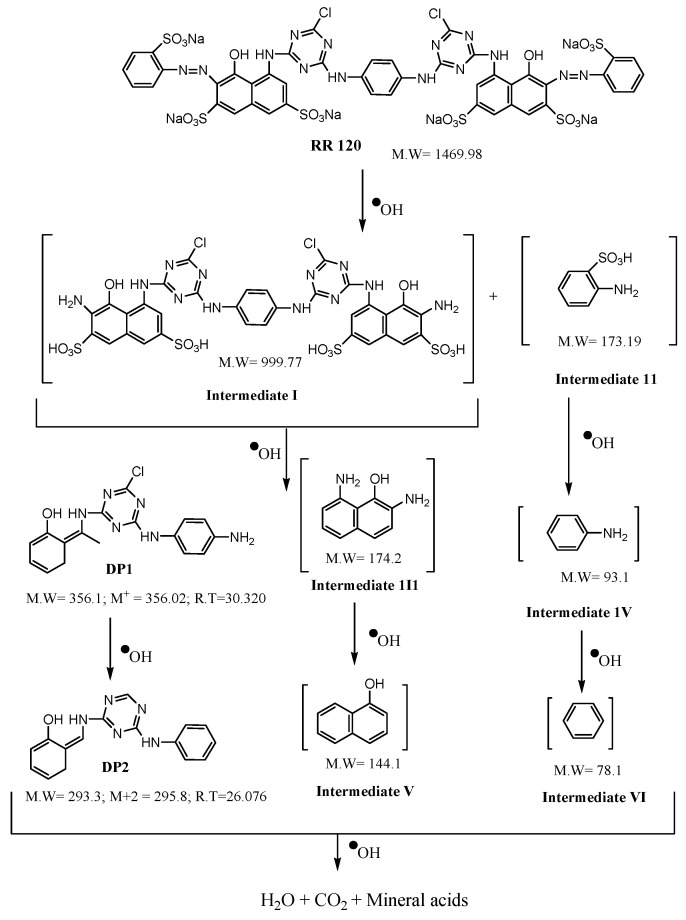
Degradation pathway of RR 120 by AC-TiO_2_.

The photocatalytic degradation pathway of RR 120 has been proposed based on GC-MS analysis (Scheme 3). During the different irradiation times, the solution was subjected to GC-MS analysis. The results were analyzed based on the molecular ion peak and fragmentation pattern, and a suitable degradation pathway was proposed. There were two degradation intermediates (DP 1 & DP 2) identified, and the details are given in Table 1. Since the degradation experiment was conducted in a slightly acidic condition (pH =5), the sulfonic acid group-containing intermediates (I and II) were expected to form via azo bond cleavage. The intermediate compound I underwent further C-N cleavage, and partial naphthyl ring-opening produced desulphonated product (6*Z*)-6-(1-(4-(4-aminophenylamino)-6-chloro-1,3,5-triazine-2-ylamino)ethylidene)cyclohexa-1,3-dienol (DP I), and naphthalene derivative (intermediate III). The intermediate compound III underwent desulfonation and produced aniline (intermediate IV). The identified degradation product DP1 further underwent dichlorination, deamination followed demethylation produced another identified degradation product (6*Z*)-6-((4-(phenylamino)-1,3,5-triazine-2-ylamino)methylene)cyclohexa-1,3-dienol (DP 2). The naphthalene intermediate III underwent deamination produced naphthalene-1-ol (intermediate V), at the same time aniline (IV) underwent deamination and produced benzene (intermediate VI). It was expected that upon continuous attack of hydroxyl radicals, the compounds DP2, V, and VI would undergo further degradation and be mineralized to CO_2_, water, and mineral acids via this advanced oxidation process (AOPs).

The efficiency of the prepared composite (AC-TiO_2_) was compared with other reported carbon-modified TiO_2_ based photocatalysts [57,58,59,60,61,62,63,64,65] (Table 2). The data were compared under different optimization conditions, and the comparison study gave the efficiency of the materials at specific reaction conditions. From Table 2, our prepared material shows a higher degradation efficiency of the RR 120 dye in a higher initial concentration (50 ppm) compared with other reported literature.

## 4. Conclusions

Biomass-derived activated carbon was prepared from pistachio shell and effectively loaded on TiO_2_ (AC-TiO_2_) for photocatalytic applications. The formed composite was utilized for RR 120 and OFL degradation under UV-A light. The prepared nanocomposite was characterized using various characterization techniques such as FT-IR, Raman, XRD, HR-TEM, FE-SEM, EDS, elemental mapping, XPS, PL, and DRS. In XRD, there were no new peaks observed for AC in AC-TiO_2_, although AC showed two broad predominant peaks. FE-SEM revealed that bare TiO_2_ is agglomerated, but the agglomeration occurs in a definite order. Spheres were formed by the aggregation of individual fine nanosized particles. The less intense PL peaks in AC-TiO_2_, when compared with bare TiO_2_, may be due to the presence of AC in the composites, which can suppress the electron-hole recombination. This may be the reason for the enchantment of photocatalytic activity of the AC-TiO_2_. The AC-TiO_2_ composite was efficient toward photodegradation of RR 120 and OFL under UV-A light compared with bare TiO_2_. The intermediates formed during degradation of RR 120 with AC-TiO_2_ under UV-A light were analyzed by GC-MS, and a suitable degradation pathway was proposed. The degradation of RR 120 occurred via the two identified intermediates (6*Z*)-6-(1-(4-(4-aminophenylamino)-6-chloro-1,3,5-triazine-2-ylamino)ethylidene)cyclohexa-1,3-dienol (DP I), and(6Z)-6-((4-(phenylamino)-1,3,5-triazin-2-ylamino)methylene)cyclohexa-1,3-dienol (DP 2). The optimum pH for the efficient removal of RR 120 was found to be 5. The photocatalyst’s stability and reusability indicated that the as-prepared material significantly influenced photocatalytic performance.

## Figures and Tables

**Table 1:** Mass spectral data of identified intermediates during degradation of RR 120 by AC-TiO_2_.

S. No.	Compounds	Name of the Compound	Retention Time (min)	M^+^/M + 1/M + 2 Values
1	DP1 (C_17_H_17_ClN_6_O)	(6*Z*)-6-(1-(4-(4-aminophenylamino)-6-chloro-1,3,5-triazin-2-ylamino)ethylidene)cyclohexa-1,3-dienol	30.320	356.0 (M^+^),
2	DP2 (C_16_H_15_N_5_O)	(6*Z*)-6-((4-(phenylamino)-1,3,5-triazin-2-ylamino)methylene)cyclohexa-1,3-dienol	26.076	295.8 (M + 2)

**Table 2:** Comparison of the efficiency of the AC-TiO_2_ composite with other carbon modified catalysts.

S. No.	Catalysts/(Dye or Pollutant) ^a^	Light Source	Concentration	Catalyst Amount	%Degradation/ Time (min)	Ref.
1	RFCA/TiO_2_/(MO)	UV light	8 × 10^−5^ mol/L	1 mg/3 mL	55/240	[57]
2	TiO_2_@carbon/(MB)	35-W Xe arc lamp/89 mWcm^−2^	1.5 × 10^−5^ M	Immersion of TiO_2_@carbon core-shell nanowires/2 mL	65/180	[58]
3	TiO_2_/MWCNT/(MB)	UV/Philips 96 W	10 ppm	0.01 g/20 mL	61.6/30	[59]
4	F-TiO_2_/C_60_@ NiFe_2_O_4_/(MG)	Halogen lamp (500 W)	30 ppm	10 mg/100 mL	98.7/120	[60]
5	F-TiO_2_/MWCNT@ NiFe_2_O_4_/(MG)	Halogen lamp (500 W)	30 ppm	10 mg/100 mL	93.5/120	[60]
6	CNT/CD/FA/TiO_2_/ (MB)	UV-C (32 W)	10 ppm	200 mg/25 mL	99/187	[61]
7	TiO_2_@carbon nanotube/(MB)	UV-C/254 nm, 2.5 mW cm^−2^	1.25µM	0.4 mg/mL	85/60	[62]
8	CX/TiO_2_/(APP)	125 W mercury lamp/80 Wcm^−2^ to 300 Wcm^−2^	25 ppm	0.1 g/50 mL	96/250	[63]
9	TiO_2_/CNDs/MIP/(PM)	LED lamps/(λ > 390 nm)	20 ppm	10 mg/100 m L	94.4/120	[64]
10	rGO/TiO_2_/BiOI/(MO)	300 W xenon lamp	50 ppm	50 mg/100 mL	94/50	[65]
11	AC-TiO_2_/(RR 120)	365 nm UV-A light/32 W	50 ppm	50 mg/100 mL	95/50	Present work
12	AC-TiO_2_/(OFL)	365 nm UV-A light/32 W	10 ppm	50 mg/100 mL	82/50	Present work

^a^ RFCA—carbon aerogel; MWCNT—multiwall carbon nanotube; CX—carbon xerogel; MO—methyl orange; MB—methylene blue; MG—malachite green; APP—acetaminophen: MIP—molecularly imprinted poly-o-phenylenediamine.

## Data Availability

All data available in the manuscript.
